# How Does Zinc Improve Salinity Tolerance? Mechanisms and Future Prospects

**DOI:** 10.3390/plants12183207

**Published:** 2023-09-08

**Authors:** Jinhua Shao, Wei Tang, Kai Huang, Can Ding, Haocheng Wang, Wenlong Zhang, Ronghui Li, Muhammad Aamer, Muhammad Umair Hassan, Rehab O. Elnour, Mohamed Hashem, Guoqin Huang, Sameer H. Qari

**Affiliations:** 1 China Guangxi Key Laboratory of Water Engineering Materials and Structures, Guangxi Hydraulic Research Institute, Nanning 530023, China; jinhua20211103@outlook.com (J.S.); slkxtw@163.com (W.T.); gxhuangkai@126.com (K.H.); dingcannn@163.com (C.D.); zwl2020520@163.com (W.Z.); 2 Research Center on Ecological Sciences, Jiangxi Agricultural University, Nanchang 330045, China; muhammadaamer@jxau.edu.cn (M.A.); muhassanuaf@gmail.com (M.U.H.); hgqjxes@sina.com (G.H.); 3 College of Civil Engineering and Architecture, Guangxi University, Nanning 530004, China; 4 Biology Department, Faculty of Sciences and Arts, King Khalid University, Dahran Al-Janoub, Abha 64353, Saudi Arabia; relgezouly@kku.edu.sa; 5 Department of Biology, College of Science, King Khalid University, Abha 61413, Saudi Arabia; mhashem@kku.edu.sa; 6 Department of Biology, Al-Jumum University College, Umm Al-Qura University, Makkah 21955, Saudi Arabia; shqari@uqu.edu.sa

**Keywords:** antioxidants, genes expression, osmolytes accumulation, photosynthesis, ROS, salinity stress, zinc

## Abstract

Salinity stress (SS) is a serious abiotic stress and a major constraint to agricultural productivity across the globe. High SS negatively affects plant growth and yield by altering soil physio-chemical properties and plant physiological, biochemical, and molecular processes. The application of micronutrients is considered an important practice to mitigate the adverse effects of SS. Zinc (Zn) is an important nutrient that plays an imperative role in plant growth, and it could also help alleviate the effects of salt stress. Zn application improves seed germination, seedling growth, water uptake, plant water relations, nutrient uptake, and nutrient homeostasis, therefore improving plant performance and saline conditions. Zn application also protects the photosynthetic apparatus from salinity-induced oxidative stress and improves stomata movement, chlorophyll synthesis, carbon fixation, and osmolytes and hormone accumulation. Moreover, Zn application also increases the synthesis of secondary metabolites and the expression of stress responsive genes and stimulates antioxidant activities to counter the toxic effects of salt stress. Therefore, to better understand the role of Zn in plants under SS, we have discussed the various mechanisms by which Zn induces salinity tolerance in plants. We have also identified diverse research gaps that must be filled in future research programs. The present review article will fill the knowledge gaps on the role of Zn in mitigating salinity stress. This review will also help readers to learn more about the role of Zn and will provide new suggestions on how this knowledge can be used to develop salt tolerance in plants by using Zn.

## 1. Introduction

Plants are exposed to various biotic and abiotic stresses that seriously affect their growth and development [1,2]. Salinity stress (SS) is a serious abiotic stress that inhibits the growth, development, and productivity of plants [3]. Globally, 20% of soils are salt-affected, and this figure is projected to increase by 30% by the end of 2050 [4]. Soil salinity has both natural and anthropogenic causes [5]. Salt deposits, sea depositions, and saline water present in soil are major natural causes of SS; however, the extent of all these causes can be increased by parent rocks [5,6,7]. Moreover, intensive agricultural practices, large use of chemical fertilizers, application of salt waters, and poor drainage are the main anthropogenic causes of soil salinity [8,9]. Salinity stress is ever-increasing owing to the continuous accumulation of salts in soils owing to the application of salty irrigation water, fertilizers, and climate change [10]. High SS is caused by high concentrations of sodium (Na^+^) and chloride (Cl^−^) that induce hyper-ionic and hyper-osmotic conditions, which in turn reduce the water and nutrients uptake and result in significant growth reduction [11]. The excessive concentration of these toxic ions (Na^+^ and Cl^−^) also decreases the photosynthetic rate, nitrogen metabolism, and chlorophyll synthesis, resulting in a substantial decrease in plant growth and development [12,13].

Salinity stress affects various physiological and biochemical processes as well as growth, development, various metabolisms, water and nutrient uptake, and photosynthetic efficiency, thus causing a serious reduction in yield [14,15]. The increased concentration of salts around plant roots causes a metabolic disorder that induces toxicity symptoms on leaves, reduces the photosynthetic capacity, and assimilates production, which results in reduced growth [16,17]. Salinity stress also reduces the length of roots and lateral root numbers [18] and interferes with gravity responses (halotropism), thereby causing disordered root morphology [19]. An excessive concentration of salts also induces reactive oxygen species (ROS) production, which damages proteins, deoxyribonucleic acid (DNA), lipids, and cellular membranes [20]. Salinity stress also damages the structure of chloroplast [21] and causes the enlargement and disordered arrangement of thylakoids [20,22]. Furthermore, SS also increases electrolyte leakage and malondialdehyde (MDA) accumulation and reduces the relative water content (RWC) of plants by disturbing water uptake [23]. However, the effects of salinity stress can vary according to the concentration of salt stress, plant species, and stage of plant growth. For instance, different authors found that salinity induced more toxic effects on salt-sensitive cultivars by affecting microtubules, causing critical destabilization of the symmetric distribution of the cell content, and disturbing cell division, leading to final cell death [24,25].

Salinity stress is affected by different factors, including climate change, rapid industrialization, and uncontrolled anthropogenic activities [26,27]. Similarly, limited rainfall, high evapotranspiration, and application of salty waters are leading to an increase in salinity stress in arid regions [27]. Therefore, it is important to devise appropriate measures to mitigate the deleterious impacts of SS on plants. Generally, plants bring changes at physiological, biochemical, and molecular levels to cope with SS [3]. However, this self-defense is insufficient to combat the negative impacts of SS. Therefore, the exogenous application of osmolytes and macro- and micronutrients is considered an important approach to overcome these toxic effects [28,29].

Among micronutrients, zinc (Zn) is an important nutrient needed for plants and humans. Zn is an essential nutrient for humans that is needed for the optimum functioning of cell-mediated innate immunity and natural killer cells [30]. Zn is needed for the cells that mediate innate immunity and was also found to reduce anemia and intestinal disturbance in humans [31]. Moreover, a shortage of Zn in humans cause skin issues, lower IQ levels, night blindness, joint pains, and hair loss [32]. Zn is also an important nutrient needed for plants, and it plays an appreciable role in mitigating the adverse effects of abiotic stresses [33]. For example, Zn supplementation not only improved plant growth but also mitigated the adverse effects of SS by improving growth, chlorophyll synthesis, and activation of the antioxidant defense system [34]. Zn also protects membranes from oxidative damage by stabilizing membrane integrity and permeability [35]. Zn works as an important component of different enzymes and stabilizers of proteins that protect plants from salinity-induced oxidative damage [36]. Zn application decreases the activity of membrane-bound reduced nicotinamide adenine dinucleotide phosphate (NADPH) oxidase and improves superoxide dismutase (SOD), catalase (CAT), and peroxidase (POD) activity, thereby inducing stress tolerance [37]. Zn also increases the expression of several genes (*GmZF351*, *OsZFP*, and *OSISAP1*) and decreases the uptake of Na^+^ by affecting the structural integrity and permeability of cell membranes [38,39]. Zn application also reduces ROS production and maintains membrane permeability, which in turn reduces the loss of important osmolytes [40]. Therefore, in this review, we systematically present the various mechanisms mediated by Zn to induce salinity tolerance. We have highlighted the conducted research and what still needs to be assessed regarding the role of Zn in SS. We believe that this review will help readers learn more about the role of Zn in SS, and it will also provide a path showing how this knowledge could be used to develop SS tolerance.

## 2. Plant Responses to Salinity Stress

Germination is the most important and crucial stage of plant life [41,42]. However, SS reduces water uptake by decreasing soil osmotic potential [43,44] and altering the synthesis of proteins and energy production [43]. Furthermore, SS disturbs the hormonal balance and nutrient uptake during seed germination, therefore delaying seed germination [45]. Salinity stress also disturbs the activity of enzymes such as α-amylase, which reduces the translocation of sugars to developing embryos [46,47]. The response to SS of a plant varies according to the stage of plant life and the intensity of the stress [48]. Generally, SS affects plant growth in two phases: in the first phase, a reduction in growth occurs in a few minutes owing to a reduction in water uptake [48].

**Figure 1:**
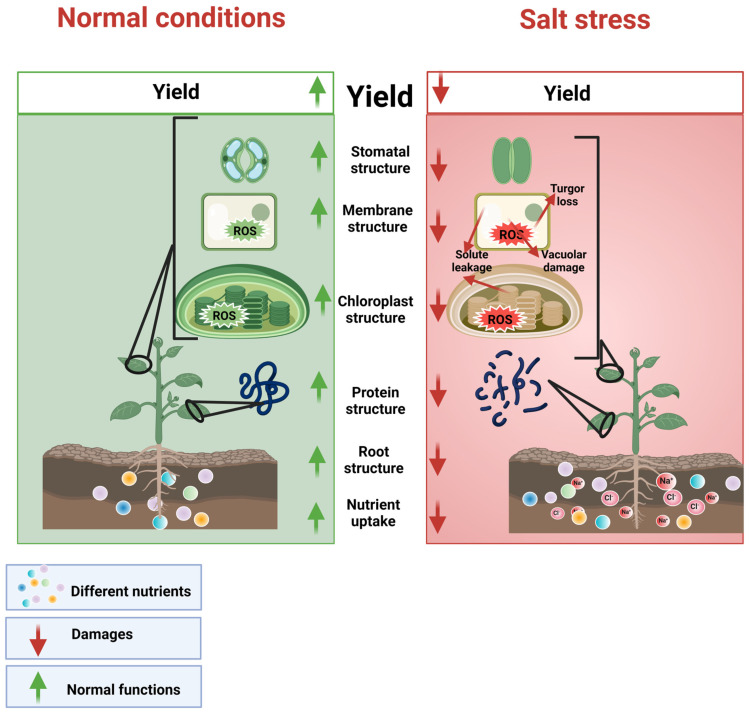
Salinity stress increases the accumulation of Na^+^ and Cl^−^, which reduces K^+^ uptake; damages membranes, proteins, and chloroplast structure; and inhibits root growth, nutrient and water uptake, stomata movements, and chlorophyll, resulting in reduced growth and final yield.

Osmotic stress induces quick closing of stomata and reduces carbon dioxide (CO_2_) assimilation, which reduces xylem pressure [49]. The decrease in xylem pressure occurs within minutes, which reduces water and nutrient movements (Figure 1), thereby causing a substantial reduction in plant growth [50]. Further plants grown under SS also face physiological and metabolic changes, including a reduction in CO_2_ assimilation and synthesis of proteins and altered leaf water status and PS-II efficiency, and all these changes lead to a significant reduction in growth [51]. In the second phase, salts accumulate in plant leaves and reach a threshold level at which they cause toxicity. The second phase is considered to take a few days up to a few weeks and even a few months [48]. During the second phase, a high concentration of Na^+^ accumulates in intracellular spaces, which inhibits the activity of various enzymes. For example, enzymes present in various compartments of the cytosol play an important role in the synthesis of starch, glycolysis, polyamine, the phenylpropanoid pathway, and the Calvin cycle. The activities of these enzymes are controlled by potassium (K^+^) concentration [52]; however, SS reduces the concentration of K^+^ in the cytosol, which negatively affects the activities of these enzymes [53]. In the second phase, plants also show chlorosis, necrosis, and reduced growth because of reduced photosynthetic activity and cell metabolism [48,54]. Salinity stress affects plant physiological and biochemical processes, which occurs due to salinity-induced osmotic and ionic stresses [48,55].

Generally, the osmotic phase happens in plants soon after the uptake of excessive salts that cause a reduction in water uptake, chlorophyll and carotenoid synthesis, and plant growth [56]. Conversely, the ionic phase is characterized by the accumulation of Na^+^ and Cl^−^, which induce ionic imbalance, leaf necrosis, and early senescence [57]. Therefore, mitigating Na^+^ uptake is considered an important strategy to improve plant resistance against SS. Salinity stress also disturbs nutrient uptake (Table 1), which induces negative impacts on plant growth. Under saline conditions, Na^+^ uptake by roots is increased whilst uptake of phosphorus (P), nitrogen (N), calcium (Ca^2+^), magnesium (Mg^2+^), and potassium (K^+^) is decreased, which disturbs ionic balance [58]. Excessive uptake of Na^+^ and Cl^−^ also induces hyper-osmotic conditions and results in a decrease in water potential, which reduces the water uptake by roots [58] and causes a substantial reduction in final yield [59].

It has also been reported to bring structural changes in the plant body and induce negative effects on plants. For instance, it was reported that salinity stress (75–150 mM) induced the production of chloroplasts and the loss of integrity of thylakoid membranes [60]. Further salinity stress also inhibited chloroplast development and starch utilization [61] and delayed the mobilization of proteins in root cells, causing a reduction in seed germination [62]. Additionally, it increased swelling of thylakoids and decreased Fv/Fm owing to an increase in ultra-structural damage, leading to a reduction in photosynthetic efficiency [63,64]. Salinity stress also induces negative impacts on chloroplast ultra-structure, which in turn reduces plant photosynthetic efficiency. For example, higher salinity toxicity (100–200 mM) caused chloroplast distortion, scattered and deformed grana lamellae, and produced swollen starch grains, which impaired the functioning of plastids [65]. In another study, it was found that under saline stress conditions, grana and inner membranes of plastids were retained well [66]. Moreover, salinity toxicity also impaired the normal function of plastids, which are considered the home of different important biochemical processes including photosynthesis, thus leading to a significant decrease in plant photosynthetic efficiency [67,68].

**Table 1:** Effect of salinity stress on growth, physio-biochemical processes, and antioxidant activities of various plant species.

Crop Species	Salinity Stress	Effects	Reference
*Brassica juncea*	200 mM	SS increased Na^+^ uptake, H_2_O_2_ production, electrolyte leakage, and antioxidant activities and reduced chlorophyll synthesis and RWC.	[68]
Lettuce (*Lactuca sativa*)	100 mM	Salt stress significantly increased MDA, H_2_O_2_, and Na^+^ accumulation and reduced K^+^, N, and P concentration.	[69]
Rice (*Oryza sativa*)	200 mM	Saline stress decreased plant chlorophyll and carotenoid contents and root and shoot growth and increased antioxidant activities and Na^+^ accumulation.	[70]
Sorghum (*Sorghum bicolor*)	150 mM	Salinity significantly increased stress markers; damaged membrane integrity; and decreased chlorophyll contents, efficiency of PS-II, and overall sorghum growth.	[71]
Maize (*Zea mays*)	100 mM	Saline conditions significantly reduced germination, α-amylase activity, germination energy, grain weight, and K^+^ contents and led to 50% emergence.	[72]
Tomato (*Solanum lycopersicum*)	120 mM	Salinity stress reduced the stem length, fruits number, flowers/plant, fruit weight, chlorophyll, and carotenoids contents.	[73]
Pea (*Pisum sativum*)	200 mM	Saline conditions reduced membrane integrity, chlorophyll contents, lipid peroxidation, photosynthetic pigments, and photosynthetic rates and activity of PS-II.	[74]
Maize (*Zea mays*)	10 dS m^−1^	High salinity stress caused a reduction in growth and physiological traits, root and shoot growth, chlorophyll contents, RWC, net photosynthesis, and uptake of Zn and K^+^.	[75]
Olive (*Olea europaea*)	200 mM	Salinity condition restricted K^+^ uptake, fructose and mannitol accumulation, growth traits, RWC, and photosynthetic pigments and increased Na^+^ uptake and ethylene production.	[76]
Wheat (*Triticum aestivum*)	12 dS m^−1^	Saline stress reduced the chlorophyll contents, chlorophyll fluorescence, grain yield, and yield components of wheat.	[77]
Oat (*Avena sativa*)	24 dS m^−1^	Salinity stress reduced growth, biomass production, plant height, chlorophyll contents, photosynthetic rate, and RWC and increased the accumulation of Na^+^ and Cl^−^.	[78]
*Brassica napus*	55 dS m^−1^	Salt toxicity reduced growth and biomass production and increased lipid peroxidation, proline synthesis, and antioxidant activities.	[79]

SS: salinity stress; H_2_O_2_: hydrogen peroxide; MDA: malondialdehyde; RWC: relative water content.

A reduction in leaf size negatively affects photosynthesis and water use efficiency and also leads to salinity-induced physiological drought [80,81]. Salinity was also found to reduce chlorophyll synthesis PS-II photochemistry and increase ROS, which reduce plant growth [82,83]. Salinity stress also increases the production of ROS that damage proteins, lipids, and DNA and induce electrolyte leakage [84]. Plants also use another mechanism including the accumulation of osmolytes and increase water uptake and antioxidant activities [85,86,87]. Moreover, salt-tolerant plants also evolve various responses such as changes in signal transduction and metabolism to cope with SS [88]. For example, the Na^+^ transporter in halophytes regulates the quantity of Na^+^ entering into xylem before going to shoots [71]. Moreover, reception of an SS stimulus in plants reduces stomata conductance by increasing abscisic acid (ABA) synthesis [89]. The production of phytohormones is also an important strategy used by plants to cope with SS. Phyto-hormones confer SS in plants by decreasing the uptake of salts, increasing antioxidant activities and photosynthesis, and leaf water status [90]. Plants have also developed excellent antioxidant enzyme (APX, CAT, POD, and SOD) defense systems which reduce reactive oxygen species production and improve plant performance under saline conditions [91,92,93].

## 3. Zn Application as an Imperative Strategy to Improve Salt Tolerance

**Figure 2:**
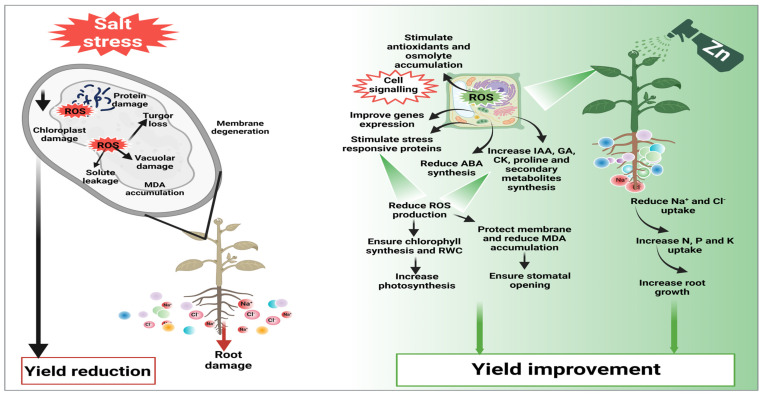
The application of Zn induces cell signaling, which increases genes expression, expression of stress-responsive proteins, antioxidant activities, and hormones and osmolytes accumulation, which reduce H_2_O_2_ and MDA and protect membranes and proteins. Furthermore, Zn also improves nutrient and water uptake and chlorophyll synthesis, maintains leaf water status, and reduces uptake of toxic ions (Na^+^ and Cl^−^), thus improving plant growth and yield under saline conditions.

Zn is a micronutrient that plays an important role in mitigating the deleterious impacts of salinity stress. Zn application induces tolerance against SS through different mechanisms, and details of these mechanisms are presented below (Figure 2).

### 3.1. Zn Application Improves Seed Germination and Seedling Growth under Salt Stress

Germination is a key phase in plant life, and SS negatively affects germination and subsequent seedling growth [94]. Salinity stress restricts plant vigor owing to osmotic and ionic toxicity and retards germination [95]. However, Zn application appreciably improves seed germination and seedling growth under SS. For instance, Zn application improves water uptake, enhances sugar accumulation and organic compounds synthesis, and modulates various mechanisms during seed germination, resulting in quick and uniform germination of seeds [96]. Germination is one of the most important processes that plays a crucial role in stand establishment and final productivity. Thus, increasing germination under SS is considered a means for the success of farmers. Edalatpishe et al. [94] noted that seed priming with Zn improved growth owing to better-developed radicals and coleoptiles in Zn-primed seeds as compared to those with no Zn priming. Additionally, Zn plays a crucial role in cellular metabolism and is involved in many proteins that favor seed germination under stress conditions [97,98]. Further, these authors also noted that grains primed with zinc sulfate (ZnSO_4_: 200 ppm) showed an improved germination speed along with higher shoot and root growth under salinity stress [98]. Recently, it has been reported that Zn nanoparticles play a beneficial role in improving the germination of seeds under SS. Likewise, it was found that zinc nanoparticles (Zn-NPs) favorably improved germination and led to hormones biosynthesis, particularly auxin and gibberellins that promote cell division and enlargement [99,100].

The Zn-mediated antioxidant capacity increases Zn contents in wheat seedlings under SS, indicating that an increased Zn concentration decreases ROS under SS [101]. Likewise, in another study, it was noted that Zn mediating plant height and biomass production was linked with increased shoot Zn concentration as compared to no Zn priming [102]. The application of Zn appreciably improves tryptophan biosynthesis, which induces a significant increase in plant growth under SS [103,104]. In another study, Zn seed priming improved protein synthesis, photosynthesis, and the activities of antioxidants (enzymatic and non-enzymatic; Figure 2), which led to a significant improvement in plant growth under saline conditions [105]. Zn supplementation also protects the cell ultra-structure and photosynthetic apparatus (Table 2) from the toxic effect of SS, which in turn improves growth and development under SS [106]. Moreover, Zn supplementation also improves protein synthesis, increases nutrient translocation from aged cells to newborn cells [107], decreases the uptake of toxic ions (Na^+^ and Cl^−^) [108], and scavenges ROS, thereby improving plant growth and development under SS [109]. Additionally, Zn supply also substantially increased photosynthetic pigments (chlorophyll) which improve photosynthesis and ensured better assimilates production, thus resulting in better growth of *Vigna radiate* under SS [110]. In conclusion, Zn application improves germination and growth under saline conditions by improving cellular metabolism and antioxidant activities and reducing ROS production.

### 3.2. Zn Improves Water Uptake and Maintains Plant Water Relations under Salinity Stress

Relative water contents (RWCs) are attributed to the structural maintenance of protein; however, SS significantly reduces the RWC [116,117]. The application of Zn allowed for maintaining a higher RWC under SS by avoiding salinity-induced water stress losses [118]. In addition, Zn application improved water uptake and mineral uptake, which led to maintaining a substantially better RWC [119]. Furthermore, Zn use maintained the RWC by improving the stability of the plasma membrane under SS [120,121]. Similarly, Sharifi and other authors also noted that the higher RWC in Zn-treated plants improved wheat production under SS [118,122]. It is believed that under SS, increased concentrations of SS inhibit nutrient and water uptake. The water deficiency collapses all metabolic processes, which impedes the ability of plants to grow; however, Zn supplementation maintains a better RWC and improves the nutrient uptake under SS by regulating the uptake and transportation of water [35]. Tavallali et al. [123] noted that reduced water uptake in *Pistacia vera* grown under saline conditions led to cellular dysfunction; however, Zn supplementation appreciably improved the water uptake and maintained the membrane stability, thereby favoring a better RWC under SS [124,125,126,127]. Thus, Zn application maintains better RWCs, which improve plant growth under saline conditions.

### 3.3. Zn Maintains Membrane Stability under Salinity Stress

Salinity-induced oxidative stress causes lipid peroxidation in membranes, reduces membrane permeability, and alters the electron transport chain by degrading proteins and impacting the repair of PSII [117,128]. Spraying saline plants (NaCl) with Zn markedly decreased the hydrogen peroxide (H_2_O_2_) and MDA concentration (Table 3), which indicates the positive role of Zn in evading oxidative damage under SS [125,129]. Salinity-induced H_2_O_2_ production and subsequent membrane damage as well as MDA formation are dependent on the intensity of salinity-induced oxidative stress [123,130]. For instance, it was reported that increasing SS increased the MDA concentration in mustard plants; however, Zn treatment effectively reduced the MDA concentration and increased membrane stability [131]. Zn is also a co-factor of different enzymes involved in ROS detoxification such as SOD enzyme [131], and it has been reported that increases in antioxidant capacity increased the Zn concentration in wheat plants, which substantially reduced ROS production and maintained membrane stability [101,102].

Zn has direct functions in the biosynthesis of plant hormones and membrane stability, and it was reported that Zn application increased membrane stability and maintained the biosynthesis of indole acetic acid (IAA) and ABA in maize seedlings under SS [102,132,133]. A salinity-induced increase in ROS production weakened the defense system of plants (barley) by overcoming the antioxidant activities of ascorbate peroxidase (APX), CAT, POD, and SOD. Nonetheless, Zn application alleviated the salinity-induced oxidative damage by improving antioxidant activities under SS [102]. In another study, Hussein and Abou-Baker [134] found that Zn mitigates SS in cotton plants by enabling higher antioxidant activities and increasing membrane permeability. Further, different authors also found that the beneficial role of Zn in maintaining biological membranes is also linked with improved antioxidant activities, cell elongation, nitrogen metabolism, and reduced ROS production [135]. In another study, it was noted that an exogenous supply of Zn reduced leaf and root electrolyte leakage (EL) through enhanced antioxidant activities, which stimulated barley growth at higher sodium concentrations [2].

Zn directly protects membranes from oxidative damage owing to the fact that it is a vital component of important enzymes, and it also stabilizes proteins’ Zn fingers [41]. Zn is a vital component of SOD and Cu/Zn isoforms that play an important role against oxidative stress. Zn supplementation decreases the activity of NADPH oxidase; reduces photo-oxidation; and increases CAT, POD, and SOD activities, thereby maintaining membrane stability under SS [123,136]. Zn has a regulatory effect on Na^+^ and Cl^−^ uptake as well as translocation rates; therefore, Zn application to salt-affected soils abates the possible injuries due to Na^+^ and Cl^−^ such as lipid peroxidation and ROS [137]. In another study conducted on mung bean, it was noted that Zn reduced thiobarbituric acid reactive substances (TBARS) and increased the stability of membranes, which indicates the positive role of Zn in alleviating ROS damage to membranes under SS [125]. In conclusion, Zn application improves membrane stability by reducing MDA and H_2_O_2_ production through improved antioxidant activities, osmolytes accumulation, and reduced Na^+^ and Cl^−^ accumulation.

### 3.4. Zn Improves Nutrient Uptake and Maintains Nutrient Homeostasis under Salinity Stress

Salinity stress significantly alters nutrient uptake, and Na^+^ is considered a primary toxic element that interferes with K, inhibits the functioning of stomata, and causes water loss and necrosis. It has been reported that Zn decreases the uptake of excessive Na^+^ and Cl^−^ and ensures efficient exclusion and increased K^+^ and Ca^2+^ uptakes that improve salinity tolerance [108]. Higher doses of Zn priming increased the Zn concentration in plant leaves, which improved the plant’s ability to tolerate salinity stress by preventing osmotic and ionic shocks [138]. Zn-mediated decreases in Na^+^ and increases in Ca^2+^ and K^+^ provide plants with a better ability to grow under saline conditions; furthermore, Zn also increases the loading of both micro- and macronutrients and the function of cellular components (mitochondria and chloroplasts) and leads to a significant improvement in plant growth and development [119].

Zn application effectively decreases Na^+^ accumulation and improves K^+^/Na^+^, which maintain membrane stability, whereas Zn deficiency induces high membrane permeability and leakage of important compounds from plants [110]. On the other hand, Zn supply also improved the uptake and concentration of K^+^, P, Ca^2+^, Fe, and Zn and Ca/Na^+^ ratio under SS, which in turn improved plant growth [110]. Generally, it is considered that Zn application reduces the deleterious effects of SS on shoots dry weight and uptake of nutrients; thus, it is recommended that if plants are grown in saline soil, especially with low Zn in the soil, a sufficient amount of Zn must be applied to the crops [111]. Moreover, these authors found a decrease in Na^+^ and an increase in N, K^+^, P, and K^+^/Na^+^ with Zn use, which led to an increase in plant growth and maintenance of membrane integrity [111].

Safari et al. [139] reported that the application of sulfur along with Zn (25 mg kg^−1^) under salinity stress increased the P, K, and Zn concentrations by 15.26%, 110.5%, and 376.6%, respectively, and reduced the Na^+^ concentration by 34.77% in plants as compared to the control treatment [139]. In another study, nano Zn increased the K^+^ concentration in plants, and maximum K^+^ was reported under no salinity with the application of 5 and 10 mg L^−1^ nano Zn followed by saline stress with the application of 10 mg L^−1^ nano Zn [140]. Foliar application of Zn and SS affects the dry weights of aerial plant parts and Fe and Mg concentrations. Hassanpouraghdam et al. [140] found that aerial dry weight and Mg concentration increased under 50 mM saline and control conditions (no salinity); further foliar-applied Zn (10 mg L^−1^) also significantly increased the Fe and Mg contents under SS [140]. Similarly, Dhanalakshmi and colleagues found that Zn application reduced Na^+^ uptake, increased K^+^ contents, and counteracted the toxic effects of SS [141]. Further, foliar Zn application is considered an important practice to increase Zn concentration, and it has been reported that foliar-applied Zn effectively increased Zn uptake and accumulation in plants and overcame salinity-induced oxidative and ionic damages [142,143]. To summarize, Zn-mediated improvements in nutrient homeostasis and reduced Na^+^ and Cl^−^ uptake substantially reduced the salinity stress effects in plants.

### 3.5. Zn Protects Photosynthetic Apparatus and Improves Photosynthesis under Salinity Stress

Salinity stress disturbs the photochemistry of chloroplasts, induces photo-inhibition, and decreases the photosynthetic ability of plants under SS [144]. The most important effect of SS is the degradation of chlorophyll because of the increase in the activity of chlorophyllase enzymes activity, though Zn is believed to reduce the activity of chlorophyllase and maintain Mg uptake, which results in a substantial increase in chlorophyll contents [111,145]. Zn application substantially increased chlorophyll contents owing to the fact that Zn is a structural and catalytic component of proteins and enzymes, and it also works as a co-factor for the normal development of pigment biosynthesis [110]. Additionally, Zn application increased photosynthetic pigments through its direct positive effect on Mg uptake owing to the fact that magnesium (Mg) plays a crucial role in chlorophyll synthesis [111].

Studies conducted on *Vigna radiata* and *B. juncea* indicated that Zn application markedly improved chlorophyll contents [110,146], and Zn also protects the sulfhydryl group, which ameliorates the decrease in chlorophyll contents [111]. Wheat plants primed with zinc oxide (ZnO) showed a marked increase in chlorophyll contents and biomass production under SS [147]. Zn supply substantially increased chlorophyll and carotenoid contents owing to the role of Zn in carotenoid synthesis, which protects photosynthetic tissues from the toxic effects of SS [111,148,149]. Additionally, Zn application enhanced the generation of chemical energy during photosynthesis, the synthesis of photosynthetic pigments, and the yield of plants [119].

Pathak et al. [103] also reported that Zn positively affected growth traits, cell ultra-structure, and photosynthetic pigments under SS and played a vital role in growth and development. Zn supplementation improves the synthesis of chlorophyll and absorption owing to better nutrient uptake and stomata movements [150]. Moreover, Zn application also increases chlorophyll fluorescence and prevents damage to the ultra-structure of chloroplasts and nuclei. In addition, Zn also regulates the function of chloroplasts and the repairing of PS-II, which improves plant photosynthesis under SS [151,152]. In conclusion, Zn application improves photosynthesis and protects the photosynthetic apparatus by increasing antioxidant activities and maintaining osmolytes and hormonal balance.

### 3.6. Zn Maintains Osmolytes Accumulation and Hormonal Balance under Salinity Stress

Osmotic adjustment is an important defense mechanism adapted by plants to cope with salinity stress effects. Osmolytes play an indispensable role in maintaining the osmotic potential of cellular and sub-cellular ions against diverse stress conditions [116,153]. Zn is an important nutrient that substantially improves the accumulation of osmolytes and enhances stress tolerance by osmotic adjustments. Zn application improves the accumulation of soluble proteins and amino acids and results in a significant increase in plant growth under SS [154,155]. Further, Zn application improves proteins and proline accumulation, thus preventing excessive ROS and oxidative damage-induced growth reductions in plants [154]. Zn is also essential for the formation of sugars and enzymes which are involved in the biosynthesis of amino acids [99]. Therefore, Zn application under saline conditions maintains the optimum biosynthesis of amino acids, which maintains the osmotic balance and protects plants from the damaging effects of SS [99].

Proline is an important osmolyte that plays a critical role in stress tolerance, and Zn application substantially improves proline accumulation, which regulates solute potential and water uptake [116,125,156]. Moreover, Zn also regulates the proline biosynthetic pathway, which alleviates the effects of salinity stress [35,119]. Likewise, other authors also noted that Zn application increased proline concentration under SS to maintain osmotic balance and protect the plants from salinity-induced toxic effects [157,158]. It is believed that SS induces stomata closing, which limits CO_2_ fixation during photosynthesis and the Calvin cycle. However, Zn supplementation maintains the tissue water potential and therefore ensures stomata opening and better CO_2_ fixation under stress conditions [159,160]. Additionally, proline also protects the functioning of diverse antioxidant enzymes which help to scavenge ROS [35,155].

Zn application improves the accumulation of different hormones that substantially improve plant performance under SS. For instance, it has been reported that Zn application increased roots and shoots of KSK-282 (24% and 22%) and Basmati-515 (34% and 28%) rice cultivars [39]. On the other hand, deficiency of Zn reduces auxin synthesis and often causes retardation in growth and development processes [161,162]. However, Zn supply is proven to be beneficial in increasing IAA synthesis, thus maintaining better plant growth under saline conditions [162]. The hyperosmotic signaling from SS triggers ABA accumulation in plants through combined activation of synthesis and inhibition of ABA degradation [163]. Interestingly, Nadeem et al. [39] reported that Zn application effectively reduced the ABA concentration in roots and shoots by 11.2% and 18%, respectively, which shows the effectiveness Zn in mitigating adverse effects in terms of better growth, membrane stability, and better K^+^/Na^+^ ratio. In another study, Chattha et al. [164] found that Zn application increased IAA and GA accumulation by 22.6 and 17.7% and decreased the ABA concentration by 19.5% under saline conditions [164]. Further, these authors also found that the application of Zn significantly improved free amino acids, soluble proteins, soluble sugars, and proline concentration. Zn is known to be a co-enzyme needed for the synthesis of tryptophan, which is the precursor of IAA formation; therefore, Zn-induced increases in IAA synthesis are linked with increased synthesis of tryptophan under SS [165]. Zn-induced increases in IAA improve root growth, which ensures better water and nutrient water uptake, thereby substantially improving plant growth under saline conditions [165]. In another study conducted on maize, the authors found that an exogenous supply of Zn mitigated the adverse impacts of SS by increasing the accumulation of glycine betaine and proline [166]. Other authors also reported that Zn supplementation improved the gibberellic acid (GA) concentration, which improved plant growth [167]. Additionally, Zn reduced the synthesis of ABA, which appreciably improved the salt tolerance [167]. However, there is no study available in the literature about the effect of Zn on trehalose, NO, and H_2_O_2_ accumulation under salinity stress. Therefore, more studies are direly needed at the metabolomic, proteomic, and transcriptomic levels on how Zn can affect ABA, IAA, SA, GA, trehalose, NO, and H_2_O_2_ accumulation under saline conditions.

### 3.7. Zn Improves Accumulation of Secondary Metabolites under Salinity Stress

Flavonoids are polyphenols, which are low molecular weight substances that play an important role in protecting photosynthesizing cells [168]. The application of nutrients significantly improved polyphenol synthesis in *Avena sativa* and resulted in improved growth [160,169]. Phenolic compounds play a critical role in ROS scavenging, and in plants (mung bean and amaranth), the concentration of phenolic compounds is increased in response to salt stress [35,170]. However, exogenous Zn (20 mg Zn kg^−1^) application also increased the concentration of phenolic compounds that improve salt tolerance [171].

Stress increases phenolic and flavonoid production, which improves growth performance by protecting plants from salinity-induced oxidative damage [172,173]. It has been reported that Zn application (300 µM) appreciably increased the flavonoid contents, which protected plants from salinity-induced ROS [174]. In another study, Ahmad et al. [125] found that Zn application substantially increased the phenol and flavonoid contents [125]. Likewise, α-diphenyl-β-picrylhydrazyl (DPPH) is an effective method to assess the concentration of radical scavenging [175]. Zn supplementation also increased DPPH activity owing to higher amounts of phenolic compounds [176]. Moreover, another factor behind the Zn-mediated increase in DPPH activity is the high amount of flavonoids [175,177]. Another group of authors also reported that ZnO application (0.24 mM) significantly increased the DPPH activity in tobacco plants [178]. Moreover, ferric reducing antioxidant power (FRAP) activity is also increased under salinity conditions, which was further increased by Zn application in strawberry plants [35,179]. In conclusion, Zn-mediated increases in secondary metabolites significantly scavenge ROS by strengthening the antioxidant defense system.

### 3.8. Zn Increases Antioxidant Activities under Salinity Stress

Salt stress increases ROS generation; however, plants also show a substantial increase in antioxidant activities to cope with excessive SS [111,136]. Zn application under saline conditions substantially increased antioxidant activities coupled with increased growth and production [180]. Zinc application increases SOD activity, which enables detoxification of more O^2−^ to H_2_O_2_. Further, Zn also increased the activity of APX (~30%), CAT (~25%), and POD (~40%), which substantially increased the potential of plants against saline stress [35,181]. Likewise, other authors also noted that Zn application stimulates the antioxidant activity of maize and *B. Juncea* plants, protecting the plants from oxidative stress [125,166]. Zn application amplified SOD activity, and it has been reported that Zn amplified the SOD activity in different crops including pistachio, sunflower, and eggplants [150,182].

**Table 3:** Effect of zinc application on oxidative stress markers, antioxidant activities, and osmolytes accumulation under saline conditions.

Crop Species	Salinity Stress	Zn Application	Effects	Reference
Rice (Oryza sativa)	100 mM	50 mg L^−1^	Zn application reduced the MDA and H_2_O_2_ accumulation of rice plants through enhanced antioxidant activities.	[181]
*Brassica juncea*	200 mM	1 mM	Zn supplementation reduced MDA and H_2_O_2_ accumulation and increased membrane stability by increasing APX, SOD, CAT, GR, AsA, GSH, and GST activity.	[125]
Spinach (*Spinacia oleracea*)	100 mM	0.3%	Exogenous Zn reduced H_2_O_2_ and MDA production through enhanced AsA, CAT, and POD activity and accumulation of soluble proteins, free amino acids, and flavonoids.	[182]
Wheat (*Triticum aestivum*)	12 dS m^−1^	40 ppm	Zn supply reduced H_2_O_2_ and MDA production and electrolyte leakage and increased POD, APX, and CAT activity and accumulation of proline, soluble sugars, amino acids, and soluble proteins.	[164]
Aloe vera (*Aloe barbadensis*)	180 mM	10 mg kg^−1^	Zn application decreased the concentration of oxidative stress markers through enhanced APX, CAT, POD, and SOD activities.	[183]
Faba bean (*Vicia faba*)	100 mM	50 mL/L	Zn application markedly reduced oxidative stress by increasing proline concentration; phenolic compounds; and CAT, APX, polyphenol oxidase, and POD activity.	[184]
Wheat (*Triticum aestivum*)	200 mM	40 mg/kg	Exogenous Zn application reduced the toxic effect of SS by increasing osmolytes accumulation and CAT, SOD, and GR activity.	[185]
Rice (*Oryza sativa*)	7.5 dS m^−1^	3.5 mg kg^−1^	Zn application improved membrane stability and reduced ROS production by increasing SOD, GR, and CAT activity; increasing IAA and GA activity, and reducing ABA synthesis.	[39]
Eggplant (*Solanum melongena*)	150 mM	20 mg/L	Exogenous Zn mitigated the adverse impacts of SS by decreasing ROS production and increasing proline, APX, POD, and SOD activity.	[137]
*Vicia faba*	150 mM	100 mg L^−1^	Zn supplementation decreased membrane damage and EL and H_2_O_2_ production through increased total phenolics; flavonoids; free amino acids; proline; GB; soluble sugars; and GSH, anthocyanin, AsA, CAT, and POD activity.	[186]

H_2_O_2_: hydrogen peroxide; MDA: malondialdehyde; APX: ascorbate peroxidase; SOD: superoxide dismutase; CAT: catalase; GR: glutathione reductase; AsA: ascorbic acid; GSH: glutathione; GST: glutathione S-transferase; IAA: indole acetic acid; GA: gibberellic acid; ABA: abscisic acid.

Catalase (CAT) is also an important antioxidant enzyme that scavenges H_2_O_2_ by converting H_2_O_2_ into H_2_O and O_2_. The activity of CAT is increased under saline conditions, and the addition of Zn further increases CAT activity, thereby increasing the potential of CAT to scavenge H_2_O_2_ [187]. In another study, Ahmad et al. [125] found an increase of ~20% in CAT activity with Zn application, whereas Wani et al. [188] found a marked increase of ~30% in CAT activity with Zn application, while other authors found a significant increase in APX activity with Zn application [99,189]. Glutathione S-transferase (GST) activity is increased under saline conditions, and Zn application also significantly increases GST activity. The Zn-induced increase in GST activity reduces ROS production through an increase in hormone synthesis and stress responses [190,191]. POD is also an important antioxidant enzyme, and Zn application also increased the activity of this enzyme under saline conditions [192]. Glutathione reductase (GR) plays an essential role against ROS through the reduction of glutathione (GSH), and it is documented that Zn application substantially increases GR activity and protects plants from damaging ROS [193].

Phenylalanine ammonium-lyase (PAL) is a critical enzyme, and SS significantly affects the activity of PAL [194,195]. The exogenous supplication of Zn improves PAL activity and alleviates salinity-induced deleterious impacts. For instance, Luo et al. [196] reported that Zn application (2 mM) increased PAL activity by 140%. PAL and tyrosine ammonia-lyase (TAL) are important enzymes involved in the biosynthesis of phenolic compounds [197]. Zn application increases PAL and TAL activity, which in turn increases ROS scavenging [198]. Zn acts as a co-factor for PAL and TAL enzymes; therefore, exogenous application of Zn upregulates the activity of these enzymes and increases ROS scavenging under saline conditions [198]. In conclusion, Zn application substantially improves antioxidant activities which improve plant performance by protecting them from deleterious impacts of salinity.

### 3.9. Zn Improves Genes Expression and Stress-Responsive Proteins under Salinity Stress

Zn application improves the expression of stress proteins and genes that increase a plant’s ability to tolerate saline conditions. For instance, an increased expression level of *BnCAM* and *BnPER* was noted with Zn application, which enhanced growth and germination [138]. In another study, exogenous foliar spray of Zn to rapeseed plants (*Brassica napus* L.) under SS caused a change in the expression of genes involved in salt tolerance. For instance, Zn application reduced the expression of *SKRD2*, *MYC*, and *MPK4* genes; however, Zn application increased the expression of the apurinic endonuclease-redox protein (ARP) and mitogen-activated protein kinase *(MAPK)* genes linked with an appreciable increase in plant physiological, hormonal, and developmental responses [199]. Further, these authors also found that Zn increased the expression of ARP and increased and decreased *SKRD2, MYC*, and *MPK4* expression, which indicates the potential role of Zn in coping with saline conditions. However, these authors also suggested that future studies must be focused on molecular effects to deeply understand Zn’s mode of action under SS and determine the optimum concentration of Zn. For instance, a low dose of Zn can exert a positive effect, while a high dose of Zn can cause toxicity [200]. Likewise, changes were noted for the induction of a 26 kDa protein and a 16 kDa protein by Razavizadeh [201] and Przymusinski [202] in canola and lupin plants. Zn supplementation improves the synthesis of proteins [203], and it also helps to bridge the amino acid residues that improve salinity tolerance [133,204]. To sum up, Zn mediates improvements in gene expression and significantly increases physiological functioning and hormonal and developmental responses, which improve plant performance under saline conditions.

### 3.10. Zn Brings Ultra-Structural Changes to Induce Salinity Tolerance

Salinity stress imposes serious damage to the cell structure; however, Zn protects plants from the toxic effects of salinity by bringing ultra-structural changes in the plant body. For instance, SS delimits the cell wall and damages the chloroplast structure; however, exogenous Zn protects plants from SS, and chloroplasts show a typical structure with well-arranged grana, thylakoid membranes, and starch grains [205]. Salt-affected plants show noticeable ultra-structural changes, including injured and smaller organelles and nucleus condensation. However, Zn application maintains the normal cell ultra-structure, with the ellipsoidal shape of chloroplasts, form, good distribution of grana, and a well-aligned internal lamellar system, and better integrity of the chloroplast structure [205]. Further, Pathak et al. [103] also reported that Zn application maintains growth traits and cell ultra-structure photosynthetic parameters under salty conditions [106,108]. Zn application also increases the concentration of photosynthetic pigments and protects plants from the deleterious impacts of SS [110,206,207]. Moreover, Zn markedly reduced damage to the ultra-structure of chloroplasts and nuclei, and it also maintained the activity of PS-II and chloroplast functioning [151]. Thus, Zn protects the photosynthetic apparatus and maintains chloroplast functioning and PS-II activities, which improve overall plant performance under saline conditions.

## 4. Zinc Application Improves Soil Properties to Induce Salt Tolerance

Salinity stress is one of the most destructive abiotic stresses that can cause substantial yield losses. However, Zn application appreciably improves soil properties that induce salt tolerance. For instance, in rice plants, it was noted that Zn application improved P and K availability, reduced the soil sodium concentration and sodium adsorption ratio, and balanced the cationic ratio, which improved the rice yield [208]. In another study, exogenous Zn application (25 mg kg^−1^ soil) with *Thiobacillus* improved macronutrient supply and nutrient uptake and reduced the harmful effects of salinity by decreasing Na^+^ uptake [139]. The application of Zn in combination with organic fertilizers improved soil porosity and organic matter nutrient uptake and decreased the exchangeable sodium percentage, which resulted in a significant increase in plant performance under saline conditions [209]. Moreover, Saad et al. [210] found that nano Zn significantly improved soil hydraulic conductivity, bulk density, and availability of N and Zn and reduced Na^+^ availability, which conferred salt tolerance in plants [210]. Apart from this, Zn application also increased the availability of Zn, which increased salt tolerance by improving antioxidant activities, physiological functioning, and hormone synthesis [104]. In the literature, limited information is available about the effect of Zn on saline soil properties; therefore, there is a dire need to conduct research on this aspect in future studies.

## 5. Zn Application Improves Plant Growth, Yield, and Quality under Salinity Stress

Salinity stress affects all plant processes, ranging from seed germination to root elongation and physiological, molecular, and biochemical responses. Salinity stress reduces plant vigor owing to a decrease in osmotic potential and ion toxicity. However, Zn application mitigates the harmful effects of SS by improving plant physiological and biochemical responses [94]. Zn application enhances sugar accumulation and modulates the metabolic processes during seed germination, which ensures better germination under SS [96]. Weisany et al. [108] noted that Zn application improves nutrient uptake, which protects plants from the damaging effects of SS. Zn also improves plant growth by increasing the synthesis of auxin, and it also activates cell division and cell elongation [211]. Further, Zn-mediated increases in growth under saline conditions are linked with better membrane integrity, phospholipids accumulation, protein synthesis, ROS scavenging, and nutrient translocation and restricted entry of Na^+^ and Cl^−^ [106,109,111,212].

Zn application also increases the synthesis of photosynthetic pigments through its positive effects on Mg, which is a crucial component of chlorophyll [111]. Zn also alleviates the toxic effects of SS by regulating the uptake and transport of water [111,213]. Zn application also increases yield by promoting nutrient uptake through leaves and roots. For instance, Zn application was found to substantially increase grain yield by 20–25% as compared to a control [143,214]. Excessive salinity has deleterious impacts on growth and yield, possibly because plants under saline conditions use energy to maintain the osmotic adjustment by reducing their growth and development. However, exogenous Zn application increases root extension, providing many effective root hairs that increase nutrient and water uptake and thus improving photosynthetic efficiency, grain, and biological yield [215]. Further, Zn stimulates cell division and cell enlargement by increasing auxin synthesis, and it also increases the accumulation of osmolytes and phenolic contents, which in turn improve plant growth under saline conditions [105].

Zn application also improves the accumulation of Zn in grain, which improves overall eating quality and grain protein contents [216]. It has been reported that the role of Zn in crop yield is linked with Zn-mediated improvements in CA activity, CO_2_ fixation, ribulose-1,5-bisphosphate carboxylase/oxygenase (RubisCO) activity, and photosynthetic capacity [217]. Further, Zn also improves the development of flowers and pollen tube formation, which improves seed production under stress conditions [15]. Seed quality is directly affected by Zn, and the positive effect of Zn on seed carbohydrate contents is linked with increased starch synthase, CA, and RubisCO activity [218,219,220]. Additionally, Zn also preserves enzymatic activity by binding the sulphydryl group and defends the disulfide formation, which increases the concentration of protein in seeds [220,221]. Therefore, Zn-mediated increases in plant growth and yield under saline conditions are linked with improved antioxidant activities, physiological functioning, hormonal balance, nutrient homeostasis, secondary metabolites accumulation, and soil properties.

## 6. Methods of Zn Application to Crops

Globally, various Zn application methods are being used to supply Zn to field crops. Seed priming, seed coating, soil application, and foliar spray are the main methods used to supply Zn to plants. The application method plays an important role in Zn availability in soil and plants; therefore, Zn application must be farmer-friendly and economical [222]. For example, seed priming (SP) is an environmentally friendly and economical method to supply Zn to crops, and Zn application by seed priming substantially improves seed productivity [223]. Soil application is also widely used globally to apply Zn to plants; however, in this method, a large quantity of Zn is applied to crops which is uneconomical, and most of the Zn applied by this method is not available to plants owing to the rapid fixation of Zn with soil particles [224]. Soil application with Zn considerably increased crop yield; nonetheless, this method is less effective at increasing grain Zn concentration [225].

Alternatively, the soil and foliar application method provides an effective solution to improve grain yield, grain quality, Zn availability, and tolerance against stress conditions [226]. Foliar application of Zn is also an effective method and significantly improves grain Zn contents and grain productivity [225]. In this method, a small quantity of Zn is applied; therefore, this method is economical, and it is also considered to be very important to reduce the deleterious impacts of stress [223,227]. Seed coating is another effective method to deliver Zn to plants, and it appreciably increases growth and yield [228]. The application of Zn as nanoparticles has recently emerged as an important approach to deliver Zn to plants. The application of Zn-NPs has been reported to increase the growth and yield of plants under a wide range of abiotic stresses [30,229,230]. Moreover, Tolay [110] found that soil-applied Zn (20 mg kg^−1^) significantly reduced the deleterious impacts of salinity stress, while Shaaban et al. [231] reported that foliar-applied Zn (300 L^−1^) effectively reduced the negative effects of salinity in canola. Additionally, Kavian et al. [183] reported that soil-applied Zn (5 and 10 mg kg^−1^) effectively mitigated the deleterious impacts of salinity in *Aloe vera* plants by increasing antioxidant activities and reducing ROS production.

Zinc fertilizers are used to prevent Zn deficiency and improve grain bio-fortification. The stage of plant growth is very important for Zn application as it plays an imperative role in improving salt tolerance and crop performance [30]. For instance, Chattah et al. [232] reported that applying Zn at the booting and milking stages was an important practice to improve rice growth, quality, and productivity. Likewise, Tuiwong et al. [233] found that foliar Zn application at the flowering and milking stages substantially improved growth and productivity. Recently, Nafees et al. [234] reported that Zn applied at two weeks of germination mitigated the toxic effects of salinity (MDA and H_2_O_2_ production) and improved the growth of wheat by increasing antioxidant activities and osmolytes accumulation. Moreover, Mushtaq et al. [235] found that pearl millet (*Panicum miliceum*) treated with Zn after 14 days of the seeding stage had improved root and shoot growth, membrane stability, genes expression, proline accumulation, and subsequently, salt tolerance.

The function of zinc (Zn) as a micronutrient involves the activity of six groups of enzymes including isomerases, hydrolases, lyases, transferases, and oxidoreductases, which improve plant performance. Furthermore, Zn also increases the activity of APX, POD, CAT, and SOD, which counters the toxic effects of salinity and improves salt tolerance [183]. In another study, Mathpal et al. [236] found that soil- plus foliar-applied Zn after 90 days of transplanting increased SOD and carbonic anhydrase activity, which in turn increased chlorophyll synthesis and grain protein concentration. Likewise, in wheat crop, soil and foliar Zn applied at the pre and post-anthesis growth stages effectively improved SOD and carbonic anhydrase activity and the Zn concentration in stems, leaves, and grains [217].

## 7. Conclusions and Future Prospects

Salinity stress negatively affects plant growth and development, changing plants’ physiological, biochemical, and molecular functioning. Zinc is an important micronutrient that improves growth and resistance against salinity stress. Zn application improves seed germination, enzymatic activities, stomata regulation, and water uptake, therefore improving plant growth and providing adaptive immunity to plants against salinity stress. Zn application also maintains membrane stability; improves nutrient uptake, nutrient homeostasis, osmolytes and hormones balance, and antioxidant activities; and protects the photosynthetic apparatus, all of which improve plant performance under saline conditions. Additionally, Zn restricts the uptake of toxic ions (Cl^−^ and Na^+^) and increases the uptake of favorable ions (K^+^), which in turn improves plant performance under saline conditions.

The information discussed in the present review highlights the role of Zn in improving salt tolerance in plants, yet many questions must be addressed in future study programs. For instance, the role of Zn in seed germination is poorly investigated, and more studies are direly needed on how Zn affects enzymatic activities and various germination mechanisms under salt stress. The role of Zn in nutrient homeostasis under saline conditions is also poorly studied, and more studies are needed to determine the effect of Zn on nutrient uptake and assess how Zn affects nutrient signaling under saline conditions. Another poorly studied topic is the effect of Zn application on hormones and osmolytes accumulation, and it is necessary to explore the effect of Zn on the accumulation of different hormones and osmolytes under salt stress. It would also be fascinating to determine the complex relationship between Zn and the accumulation of hormones to discover if Zn directly or indirectly enhances the synthesis of hormones and osmolytes to counter the effects of salinity stress. Moreover, the effect of Zn on osmolytes (GB, trehalose), polyamines, polyphenols, and hormones (ethylene, jasmonic acid) has not been studied comprehensively; therefore, it is necessary to explore the role of Zn here for a better understanding of its contributions against saline conditions. More studies must also be undertaken to understand the effects of Zn on gene expression and stress-responsive proteins under salinity stress. Additionally, detailed studies are demanded to understand the effect of Zn on the expression of aquaporins and their role in water uptake under salinity stress. Lastly, pilot plot studies are needed to optimize the rates and methods of Zn application for different crops on the basis of climate, plant, and soil conditions.

## Figures and Tables

**Table 2:** Effect of Zn application on growth and physiological characteristics of diverse plants under saline conditions.

Crop Species	Salinity Stress	Zn Application	Effects	Reference
*Vigna radiata*	200 mM	250 µM	Zn application offset the negative effects of SS and improved chlorophyll and carotenoid contents; soluble sugars; soluble proteins; antioxidant activities; and proline, phenol, and flavonoids accumulation.	[35]
Soybean (*Glycine max*)	99 mM	10 µM	Zn application improved root and shoot growth and biomass production; increased the uptake of K^+^, Ca, Mn, Ca, P, and Zn; and reduced the uptake of Na^+^.	[108]
Rosemary (*Salvia rosmarinus*)	120 mM	20 mg kg^−1^	Zn application decreased electrolyte leakage and shoot Na^+^ and increased CAT activity; phenolic compounds; and concentrations of Ca, K^+^, Mg, and Zn.	[109]
Barley (*Hordeum vulgare*)	100 mM	100 mg kg^−1^	The exogenous supply of Zn improved barley growth, photosynthesis, and chlorophyll contents and reduced electrolyte leakage, MDA, and H_2_O_2_ production.	[2]
Basil (*Ocimum basilicum*)	1.5% NaCl	10 mg kg^−1^	Zn supplementation increased the biomass production, chlorophyll contents, K^+^ uptake, and chlorophyll index of plants.	[110]
Tomato (*Solanum lycopersicum*)	160 mM	30 μM	Zn supply alleviated the deleterious impacts of SS by increasing solute accumulation, RWC, soluble sugars, photosynthetic activity, and antioxidant activity and reducing Na^+^ accumulation.	[111]
Almond (*Prunus dulcis*)	90 mM	20 mg kg^−1^	Zn application substantially improved photosynthetic rate, stomata conductance, PS-II efficiency, proline contents, and activity of CAT to counter SS effects.	[112]
*Brassica campestris*	160 mM	25 μM	Zn supplementation enhanced the gas exchange characteristics, biomass, root and shoot growth, transpiration, and stomata conductance.	[113]
Cotton (*Gossypium herbaceum*)	15 dS m^−1^	50 kg ha^−1^	Zn application improved the chlorophyll index, photosynthetic rate, transpiration, vapor pressure, and stomata conductance.	[114]
Rice (*Oryza sativa*)	70 mM	15 mg kg^−1^	The Zn supply improved the crop growth and biomass by increasing stomata conductance, photosynthesis and transpiration, Zn concentration, and K^+^ /Na^+^.	[115]

SS: salinity stress; Zn: zinc; RWC: relative water content; CAT: catalase; H_2_O_2_: hydrogen peroxide; MDA: malondialdehyde.

## Data Availability

Not applicable.
